# Increasing Burden of Acute Hepatitis A among Ethiopian Children, Adolescents, and Young adults: A Change in Epidemiological Pattern and Need for Hepatitis A Vaccine

**DOI:** 10.4314/ejhs.v32i2.5

**Published:** 2022-03

**Authors:** Abate Bane, Amir Sultan, Rabia Ahmed

**Affiliations:** 1 Adera Medical Center, Addis Ababa, Ethiopia; 2 Addis Ababa University, College of Health Sciences, Department of Internal Medicine, Addis Ababa, Ethiopia

**Keywords:** Hepatitis A, Expanded Program on Immunization

## Abstract

**Background:**

Hepatitis A is a vaccine-preventable, feco-oral infection due to poor sanitary conditions. It is predominantly acquired during early childhood and results in lasting acquired protective immunity. However, it results in severe disease which can end up in acute fulminant hepatitis and hepatic failure when acquired during adolescence and adulthood. The prevalence of acute hepatitis A is increasing among children, adolescents, and young adults from higher-income households. They acquire this infection at a later age when they are exposed for the first time to contaminated food and drinks after being brought up in a relatively clean environment. This calls for the introduction of the Hepatitis A vaccine in Ethiopia; possibly as part of the Expanded Program on Immunization (EPI).

**Methods:**

Socio-demographic and clinical data were collected from patients who were diagnosed to have hepatitis A infection at Adera Medical Center in 2020.

**Results:**

This study showed that clinical acute hepatitis A is becoming common among children, adolescents, and young adults from relatively high-income families. Among patients with acute hepatitis, 89% were from middle and high-income families.

**Conclusions:**

There is a need for the incorporation of hepatitis A vaccine in the Ethiopian EPI program.

## Introduction

Hepatitis A virus is a vaccine-preventable, RNA virus. Its transmission is primarily via the feco-oral route, by ingestion of contaminated food and drink (1). Poor socioeconomic status and sanitary conditions predispose to this infection during early childhood. At this age, the infection is usually mild, asymptomatic, and results in lasting acquired immunity (2). However, children from higher socioeconomic class families are raised without early childhood exposure to HAV because they are brought up in a relatively clean environment. Hence, when they are exposed for the first time through foods or drinks contaminated with HAV at schools, cafes, restaurants, or colleges they can develop an acute hepatitis A infection.

Although there were seroprevalence studies done in the past, there are no descriptive studies on acute hepatitis A infection in Ethiopia (2,4). Thus, it is essential to conduct studies on the prevalence of hepatitis A infection and its association with the socioeconomic status of the individuals in Ethiopia. This is the first descriptive study on clinical acute hepatitis A carried in Ethiopia to assess its pattern and the need for the incorporation of HAV vaccine in Expanded Program on Immunization (EPI). The objective of this study is to identify the pattern of hepatitis A infection and the socio-economic status of individuals and the need for the inclusion of HAV vaccine into the Ethiopian Expanded Program on Immunization (EPI).

## Methods and Patients

All patients who presented to Adera Medical Center(which is a private medical facility and most of the patients are from middle to high-income families) in Addis Ababa with acute hepatitis over one year from January 1, 2020, to December 31, 2020, were tested for HAV, HBV, HCV, HIV, AST, and ALT using standard laboratory methods. After informed consent and institutional approval were obtained, demographic data, clinical features, and socioeconomic data were collected from patients and/or attendants using a prepared questionnaire. Additional data on water source, sanitation, socioeconomic status, laboratory investigations, and abdominal sonography data were also gathered for those who were positive for anti-HAV IgM test. Socioeconomic class was defined as high if the family's average yearly income is greater than $8179.37, middle if the family's average yearly is between $1414.43 and $8179.37 and low if the family's average yearly income was below $1414.43 (currency exchange on August 3, 2021, 1 USD = 44.3791 Ethiopian birr) (4).

The patients with acute hepatitis were provided with standard medical treatment and follow-up with a Hepatologist at Adera Medical Center. And all of them recovered fully without any severe complications.

## Results

**Figure 1:**
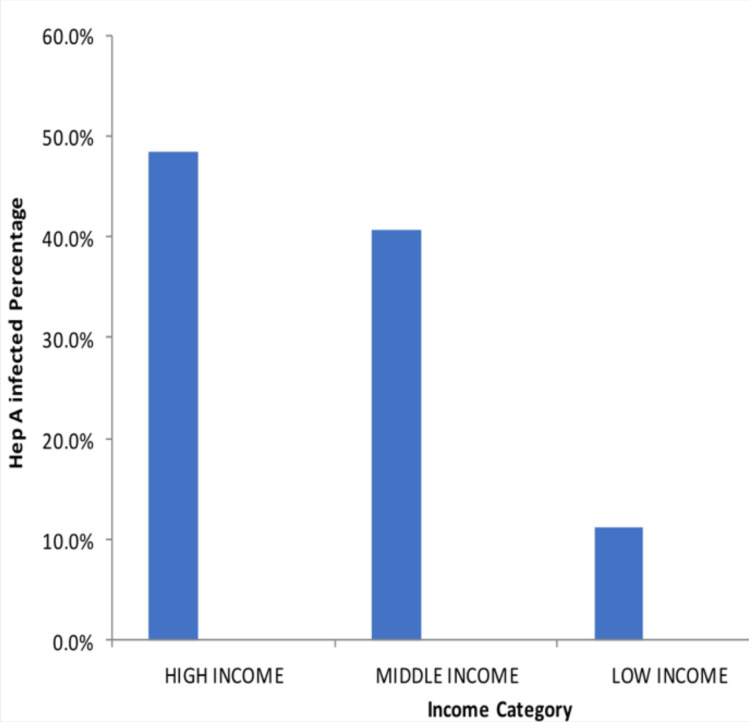
Chart representing the income of households of patients with acute hepatitis A infection at Adera Medical Center, 2020, Addis Ababa

The study was conducted among 27 children, adolescents, and young adults infected with hepatitis A virus in 2020, who presented to Adera Medical Center. Most of the patients were in the age group 15–20 and 10–15 consisting of 41% and 33% of the patients, respectively. While 2 (7%) of the patients were in the age group below 5. Of these patients, 15 (56%) were males while 12 (44%) of them were females. Among which 21 (78%) were from Addis Ababa and 6 (22%) reside elsewhere in Ethiopia. From these patients 13 (48%) were from high-income families, 11 (41%) were from middle-income families, and only 2 (11%) were individuals from low-income families (Figure 1). From the patients, 23 (85%) of them were students while 2 (7%) were below the age of 3 and the other 2 (7%) were adults who were merchants (Table 1).

**Table 1:** Socio demographic data of patients with acute hepatitis A infection at Adera Medical Center, Addis Ababa, 2020

Variables		Number	Percentage
Address	Addis Ababa	21	78%
	Out of Addis Ababa	6	22%
Sex	Male	15	56%
	Female	12	44%
	0–5	2	7%
	5–10	3	11%
Age	10–15	9	33%
range	15–25	11	41%
	above 25	2	7%
	High income	13	48%
Income	Middle income	11	41%
	Low income	3	11%

Only one of the two adult patients had a history of alcohol intake. All patients had access to pipe water and use only water from pipe water. Among the patients, 14 (52%) had onset of disease in less than a week from the time of presentation, while 13 (48%) presented more than a week from onset of symptoms.

**Figure 2:**
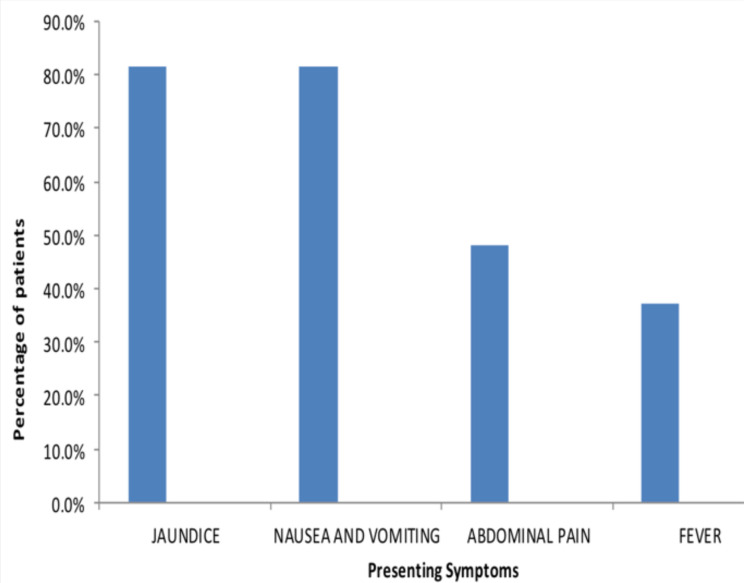
Chart representing the symptoms of patients with acute hepatitis A infection at Adera Medical Center, 2020, Addis Ababa

The most common symptoms among patients were nausea with vomiting and yellowish discoloration of the eyes and skin. Each was present in 81% of the patients. Other presenting symptoms were abdominal pain, fever, and lethargy, which were present in 48%, 37%, and 4% of the patients, respectively (Figure 2) (Table 2).

**Table 2:** Clinical presentation of patients with acute hepatitis A infection at Adera Medical Center, Addis Ababa, 2020

Sign and symptoms	Number	Percent
Jaundice	22	81 %
Nausea and Vomiting	22	81%
Abdominal Pain	13	48%
Fever	10	37%
Hepatomegaly	10	37%
Lethargy	1	4%

The most common finding on physical examination was icterus present in 85% of the patients followed by hepatomegaly identified in 37% of them.

None of the patients had any history of jaundice previously, nor did they have history of contact with a jaundiced individual. None of the patients presented with rash, joint pain, abdominal tenderness, or any other extrahepatic manifestations. None of the patients reported having history of drug usage, raw meat consumption, or vaccination for hepatitis A.

All individuals tested positive for Anti HAV IgM and were negative for HBsAg, HCV and HIV infection. On ultrasound examination 48% of the patients had normal sonographic findings, while, 26 % had hepatomegaly and 26 % had other sonographic findings like fatty liver.

Laboratory investigation results showed AST, ALT, total and direct bilirubin elevation five times the upper normal limit in majority of the patients. While ALP was less than two and a half times elevated from the upper normal limit in the majority of the patients. Leukocytosis was detected in only two patients. Hemoglobin and platelet results were normal in all the patients (Table 3).

**Table 3:** Laboratory investigation results of patients with acute hepatitis A infection at Adera Medical Center, Addis Ababa, 2020

Laboratory investigation		Number of patients	percentage
AST	Below 2.5* elevated	2	7%
	Between 2.5* and 5* elevated	9	33%
	Above 5* elevated	16	59%
ALT	Below 2.5* elevated	8	30%
	Between 2.5* and 5* elevated	1	4%
	Above 5* elevated	18	67%
ALP	Below 2.5* elevated	17	63%
	Between 2.5* and 5* elevated	9	33%
	Missing	1	4%
Bilirubin Total	Below 2.5* elevated	4	15%
	Between 2.5* and 5* elevated	9	33%
	Above 5* elevated	14	52%
Bilirubin Direct	Normal	1	4%
	Below 2.5* elevated	2	7%
	Between 2.5* and 5* elevated	3	11%
	Above 5* elevated	21	78%

## Discussion

WHO doesn't recommend routine vaccination against hepatitis A in high endemic settings, like Africa and Asia, associated with poor sanitary conditions (6). However, due to the current socioeconomic development, a growing proportion of the population is no longer exposed to hepatitis A during early childhood, hence, would not acquire lasting immunity. In the absence of HAV vaccine, this leads to acute hepatitis with severe complications when HAV infection is acquired later in life.

A seroprevalence study conducted in Ethiopia in 1983 reported the seroprevalence of HAV among blood samples from Ethiopian adults was 99% (2). Another Age-specific seroprevalence study conducted in 2009 revealed 50% HAV antibody seroprevalence among children while almost all individuals were positive for anti HAV antibody by the age of 15 (4).

In contrast to this, our study reveals that clinical acute hepatitis A is becoming common among children, adolescents, and young adults from relatively high-income families. In this study, among patients with acute hepatitis, 89% were from middle and high-income families. In addition to that most of the patients (78 %) lived in the capital city of Ethiopia, Addis Ababa. This suggests that there is an increase in the incidence of acute hepatitis A infection among children, adolescents, and young adults, who had no exposure to hepatitis A virus, because of their living in a relatively sanitary environment and therefore, have not developed immunity in early childhood. This is in agreement with studies from other developing countries in Africa, Asia, and Latin America (6,8,9,10).

A study carried out in 2015 in Africa concluded that with the current strategy of no HAV vaccination in EPI program, Africa sits on an impending HAV epidemic if epidemiology of the disease is not well understood and appropriate measures for control are not adopted and implemented. Moreover, there is no systematic evidence that shows HAV infection trends limit the adjustment of current policies on control and prevention of hepatitis A infection (6).

Subsequent studies done recently in Africa in 2019 suggest that epidemiological data that is currently missing should be compiled and priority be given to re-assessing the current hepatitis A control strategies in the region to prevent possible disease outbreaks in the future (8).

A study conducted in India in 2019 also revealed that due to current shifts in endemicity, a growing proportion of the population is no longer exposed in childhood to HAV. As the disease remains highly endemic, it also provides a source for more severe disease in susceptible people at an older age and for outbreaks (8).

Another study done in Latin America in 1999 suggested that the epidemiology is shifting from high to intermediate endemicity, with the population susceptible to HAV infection shifting from children to adolescents and adults. Furthermore, data from Brazil, Argentina, and Mexico showed that HAV seroprevalence is significantly lower in people living in medium and high socioeconomic conditions. This study suggested the need for appropriate vaccination programs to be implemented targeting children, adolescents, and adults, particularly in higher socioeconomic groups (9).

Given the growing socioeconomic development and projected advancement in better sanitary living situations, acquisition of HAV infection in later life stages will be common at which time the disease will be severe and complicated with hepatic failure and even death. This will also have a significant economic burden on families and health institutions in added time lost from school, colleges, and workplaces. Fortunately, well-tolerated and effective vaccines are available and help prevent disease burden and provide long-term protection. A study conducted in 2003 on the efficacy of HAV vaccine showed that combined inactivated vaccine effectiveness was 86% (95% CI: 63–95%). Combined attenuated vaccine effectiveness was 95% (95% CI: 81–99%) (10). These should now be used more widely to protect the population from the growing disease burden of hepatitis A.Therefore the introduction of an effective HAV vaccine in the Ethiopian Expanded Program on Immunization (EPI) vaccination program is timely, cost-effective, and lifesaving.

In conclusion, as the socioeconomic status of Ethiopia is improving (11), the prevalence of acute hepatitis A infection appears to be shifting towards adolescents and young adults. This is partly due to reaching adolescence and adulthood without acquiring immunity from early childhood infection. Thus, there is a rationale to make hepatitis A vaccine available for vulnerable groups immediately and to incorporate hepatitis A vaccine in the Expanded Program on Immunization (EPI) in the future to combat the spread of HAV as well as the complications associated with acute hepatitis A infection.

We also suggest that there is a need to do a wider population-based study and increase awareness of viral hepatitis in general and specifically on hepatitis A. Increasing awareness on community and individual hygiene as well as regular medical checkups are also essential preventive strategies.
